# Ultrasound-guided surgery for lateral snapping hip: a novel ultraminimally invasive surgical technique

**DOI:** 10.1186/s13018-021-02461-y

**Published:** 2021-05-19

**Authors:** Manuel Villanueva, Álvaro Iborra, Pablo Sanz-Ruiz, Concepción Noriega

**Affiliations:** 1 Avanfi Institute and Unit for Ultrasound-guided Surgery, Hospital Beata María Ana, Calle de Donoso Cortes 80, 28015 Madrid, Spain; 2 Orthopaedic and Trauma Department, Hospital General Universitario Gregorio Marañón, Madrid, Spain; 3 Department of Nursery and Physiotherapy, Faculty of Medicine and Health Sciences, University of Alcalá, Alcala de Henares, Madrid, Spain

**Keywords:** Ultrasound-guided surgery, Ultrasound, Snapping hip

## Abstract

**Background:**

Greater trochanteric pain syndrome encompasses a range of causes of lateral hip pain including greater trochanteric bursitis, tendinopathy and tears of the gluteus minimus and medius, and lateral snapping hip (LSH).

Surgical options for LSH range from open surgery to endoscopic surgery, including a diamond-shaped cut or a simple transversal release to gluteus maximus tendon release.

Resection of an area of proximal iliotibial band (ITB) and step-cut or z-plasty lengthening have not proven superior to transverse release of the ITB. Therefore, making a complete and effective transverse cut guided by ultrasound may represent a potential advance over endoscopic surgery.

**Purpose:**

In this case series study, we describe how to perform proximal release of the ITB guided by ultrasound.

**Methods:**

The surgical technique—either z-plasty or transverse section of the ITB—was first validated on 10 cadaver specimens and then used in clinical practice. Fourteen patients (5 males and 9 females) were operated from 2014 to 2018. Mean age was 43 years (29–62).

**Results:**

The snap resolved in all patients, as verified actively during the surgical procedure as the patient has only local anesthesia. The VAS score for sports activity improved from 7 (5–9) before surgery to 0 (0–2) after 1 year. The HSS score improved from 58 points (47–72) to 96 at 1–2 years. There were no complications other than minor hematomas nor recurrences.

**Conclusion:**

Ultrasound-guided release of the LSH is a novel surgical option with encouraging results in patients for whom conservative protocols have failed. It can be performed under local anesthesia in an outpatient setting with minimal aggressiveness. It is relatively easy, quick, and painless; no stitches are required. Weight bearing is immediate, and patients usually need crutches for only 2–3 days. Although complete recovery may take 3 months, the rehabilitation protocol is fast and painless.

## Introduction

Approximately 5 to 21% of all sports injuries involve the hip and pelvis. Data from a general sports medicine clinic show that overuse accounted for 82.4% of the injuries to the hip and pelvis [1–3].

Greater trochanteric pain syndrome (GTPS) encompasses a range of causes of lateral hip pain including greater trochanteric bursitis, tendinopathy and tears of the gluteus minimus and medius, and lateral snapping hip (LSH) [3–6]. These conditions can be managed with surgical repair and release of the iliotibial band (ITB) [6, 7].

For most people, the only symptom is the snapping sound or the sensation itself. However, for dancers and athletes, it can cause pain in the area of the trochanter that can radiate along the lateral border of the thigh, leading to weakness that interferes with performance. The pain is usually aggravated with sports and lying on the affected side [8–10]. LSH is associated with a tight ITB.

Surgical options for LSH range from open surgery—including resection combined or not with partial reconstruction of the posterior flap (70–90% success rate) [11–14] and step-cut or z-plasty lengthening (30–100% success rate) [15–21]—to endoscopic surgery. Endoscopic surgery techniques range from creating a cross-shaped cut plus a diamond-shaped cut [22, 23] and a simple transversal release [24] to gluteus maximus tendon release [25]. These techniques have a success rate of 90–100% [24–28].

Surgical techniques based on resection of an area of proximal ITB and step-cut or z-plasty lengthening have not proven superior to transverse release of the ITB, as first described by Dickinson [12] in 1929 and later by Gordon [29]. Therefore, the idea of making a complete and effective transverse cut guided by ultrasound may represent a potential advance over endoscopic surgery.

Ultrasound-guided surgery is a novel approach with proven indications, such as gastrocnemius lengthening, carpal tunnel release, tarsal tunnel release, plantar fasciitis, and distal iliotibial band syndrome [30–35].

In this study, we describe how to perform transverse release of the proximal ITB guided by ultrasound. The surgical technique—either Z-plasty or transverse section—is theoretically easier than other ultrasound-guided approaches. To the best of our knowledge, this is the first report of ultrasound-guided surgery for LSH.

## Material and methods

Our prospective study was performed in accordance with the principles of the 1964 Declaration of Helsinki (2013 revision) and approved by the Research Ethics Committee of Hospital Beata María, Madrid, Spain. All participants gave their informed consent to participate and for their clinical and radiological data to be reproduced. The Anatomy Department of Francisco de Vitoria University, Pozuelo de Alarcón (Madrid), Spain, provided the specimens used in this study. Surgical studies using specimens from body donors do not require ethics committee approval.

We performed a pilot study with 10 cadavers to ensure that the technique was effective and safe for complete release of the proximal ITB without damaging other structures. In a second phase, between May 2014 and May 2018, data from a prospective clinical series of 14 patients (5 males, 9 females) were included. Inclusive criteria were failure of conservative protocol after at least 6 months before surgery. Patients were limited with respect to sporting activity but not activities of daily living, despite pain and discomfort.

Resolution of the snap was checked immediately during surgery under local anesthesia, and movement of the leg was actively encouraged. We were thus able to verify that the snap had resolved.

The results were evaluated based on a visual analog scale (VAS) and the Harris Hip Score (HHS) [36]. A VAS was also administered to assess pain, degree of satisfaction, and return to normal activities at 3 months, 6 months, 1 year, and 2 years. The questionnaires evaluated restrictions in daily activities, which for most included sports activities.

The statistical analysis was performed using R Ver. 5.3.1 (R Foundation for Statistical Computing, Institute for Statistics and Mathematics, Welthandelsplatz 1, 1020 Vienna, Austria). Statistical significance was set at *p* <0.05. Qualitative variables were expressed as absolute values and frequencies; quantitative variables were expressed as mean and standard deviation. Outcomes for VAS and the HHS were analyzed using the omnibus Friedman test with post hoc tests and a Wilcoxon signed-rank test with a Bonferroni correction. The effect size was defined with Kendall’s *W* as small (<0.1), medium (0.10–0.25), and large (>0.25).

### Surgical technique

**Fig. 1:**
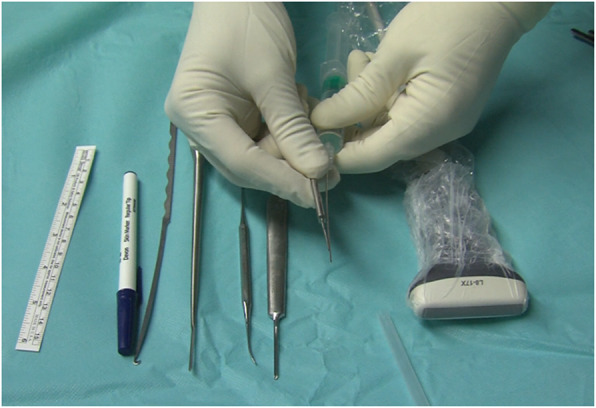
Instrument set

The instrument set includes long needles, a V-shaped straight curette, a blunt dissector, a hook knife, and an ultrasound device with a 10- to 17-Mhz linear transducer (Fig. 1).

**Fig. 2:**
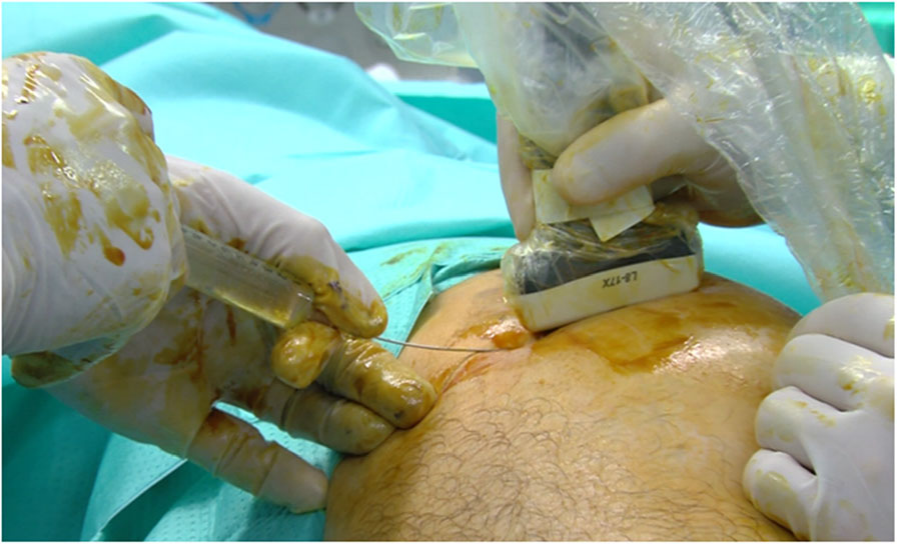
Ultrasound-guided insertion of the guide needle

**Fig. 3:**
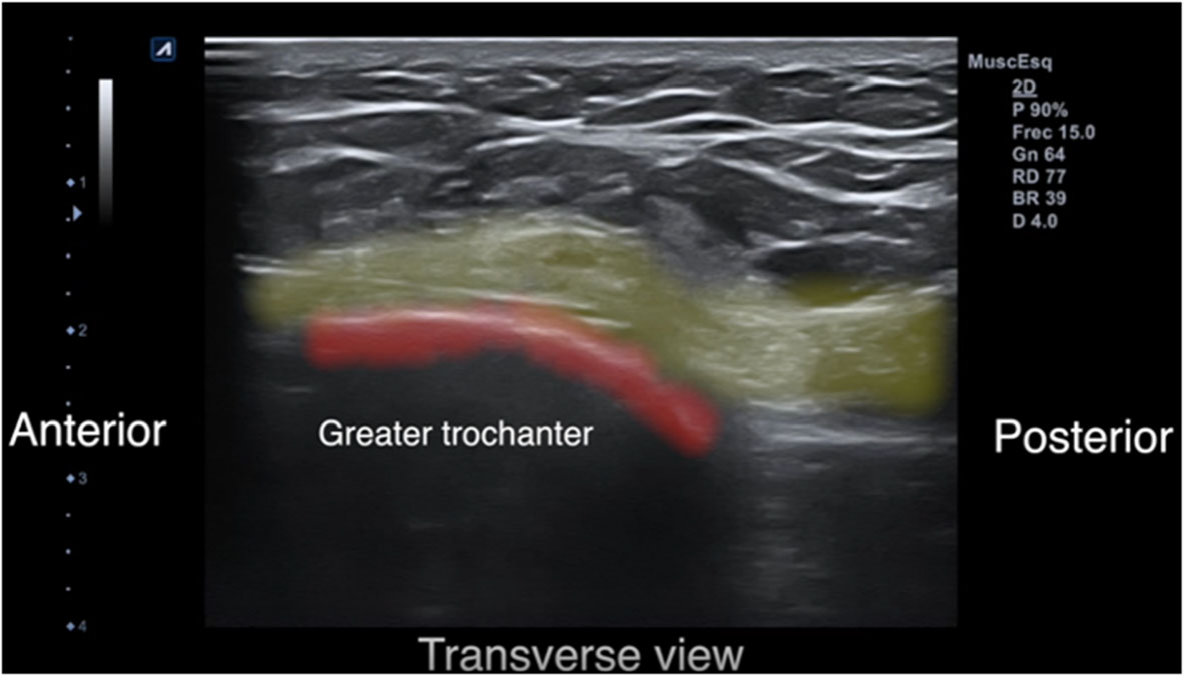
Needle inserted beneath the ITB (yellow band)

**Fig. 4:**
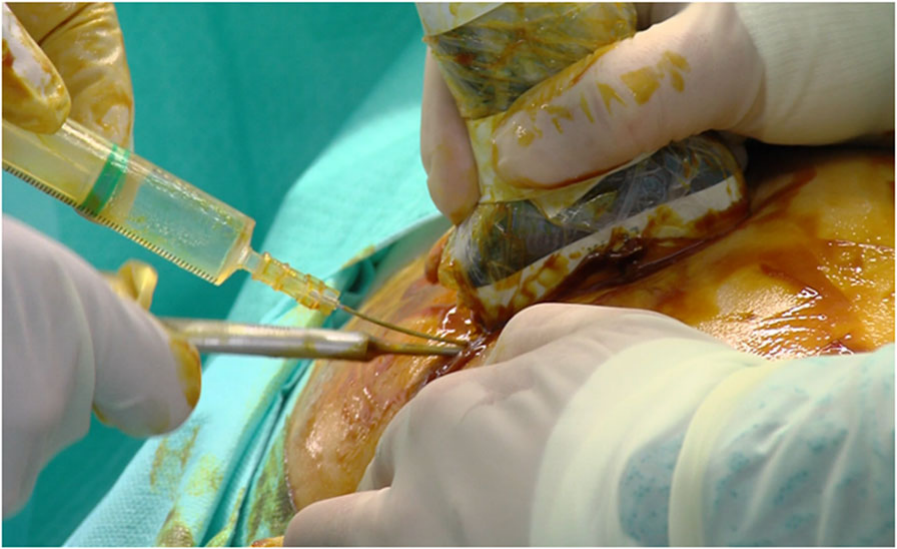
The needle guides the insertion of the first gauge

The patient is placed in the lateral or the supine position. We use local anesthesia plus sedation, when necessary. First, with the probe in the longitudinal axis, we locate the selected point for the recession. This is at the mid-point of the greater trochanter or slightly proximal to it. We then create an acoustic shadow with the blunt dissector. Next, we create marks on the skin with the hollow tip of a syringe to define the entry point and the direction of the recession. At the selected point, we place the probe in the transverse axis and identify the ITB over the greater trochanter. Using continuous ultrasound guidance, 8–10 cc of 1% mepivacaine is injected as the needle is guided down the ITB from anterior to posterior (Figs. 2 and 3). Thus, we create a working space beneath the ITB. It is important to reach the limit of the gluteus muscle to avoid tension on unreleased residual fibers of the ITB. We insert 2 V-shaped straight curettes (small and medium) to enlarge the entry point and seal the entry point with betadine gel to prevent air bubbles from entering and distorting the ultrasound image (Fig. 4). We then insert the hook knife following the curve of the blade so as not to enlarge the incision. If this proves difficult, we repeat the gesture with the larger v-shaped curette.

**Fig. 5:**
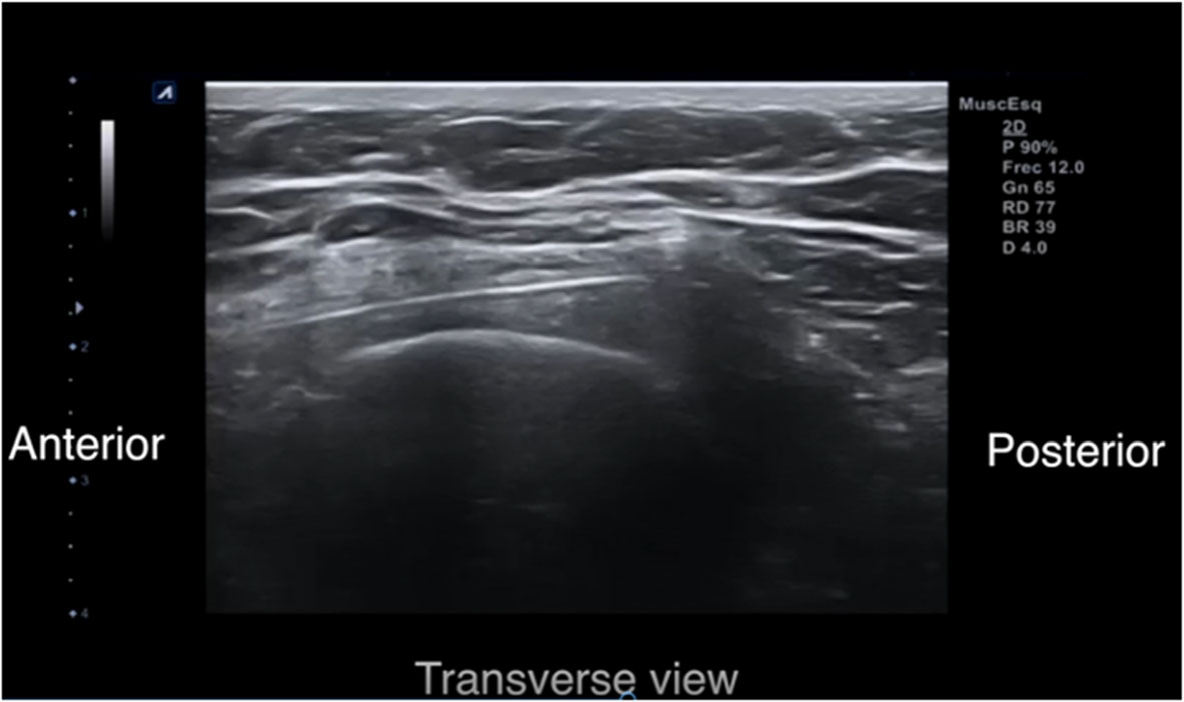
The hook-knife reaches the posterior limit of the proximal ITB. The fibers of the muscle are clearly different from the ITB. Ultrasound image

**Fig. 6:**
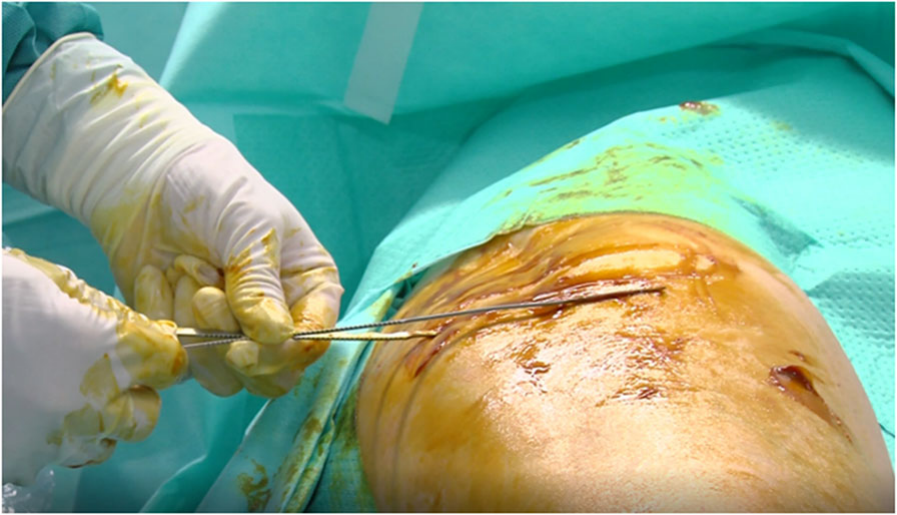
The hook knife reaches the posterior limit of the proximal ITB. The other knife illustrates the posterior limit

**Fig. 7:**
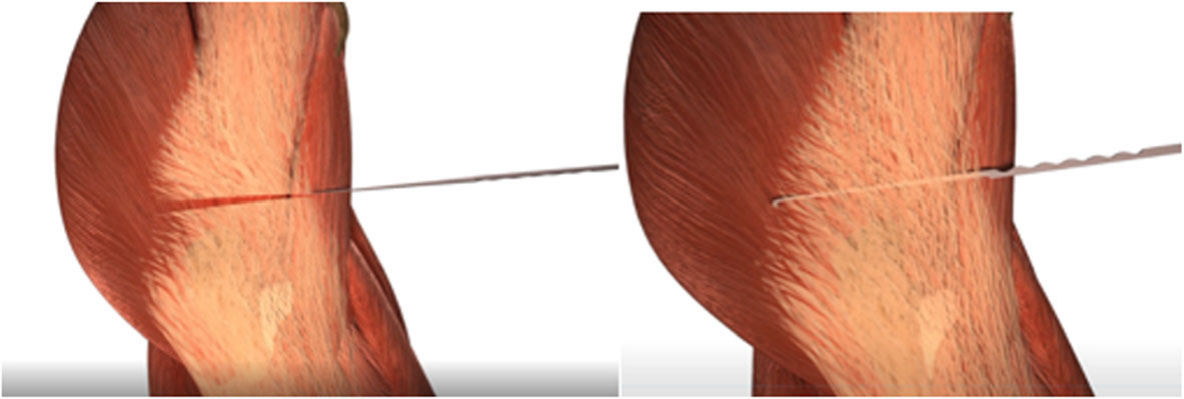
The hook knife must reach the posterior limit of the ITB, the anterior limit of the gluteus maximus muscle

With the transducer placed in the transverse position, we advance the hook-knife in the horizontal plane towards the posterior border of the ITB until we reach its posterior limit. Reaching the limit of the gluteus maximus, where it meets the ITB, is the critical point for complete release of the structure and resolution of the snapping hip (Figs. 5 and 6). Incomplete release of the most posterior fibers of the ITB may lead to failure of the procedure (Fig. 7). The blade is then turned 90° towards the tendon. We can pull the knife backward and forward until the tip of the hook severs the fibers of the fascia lata and is in a perfect position to start the retrograde release. At the posterior border of the fascia lata, we start severing the tendon in a posterior to anterior direction.

We always perform the procedure with 2 surgeons, one of whom can concentrate on accurate positioning of the instruments. The surgeon pulls the hook-knife back with both hands, with at least one resting on the patient’s thigh or on the operating table to control movement and maintain the direction of the cut. The second surgeon holds the probe and stretches the ITB by adducting the leg. If necessary, we repeat this gesture 2–3 times. We remove the hook-knife following the curve of the hook so as not to enlarge the entry point.

**Fig. 8:**
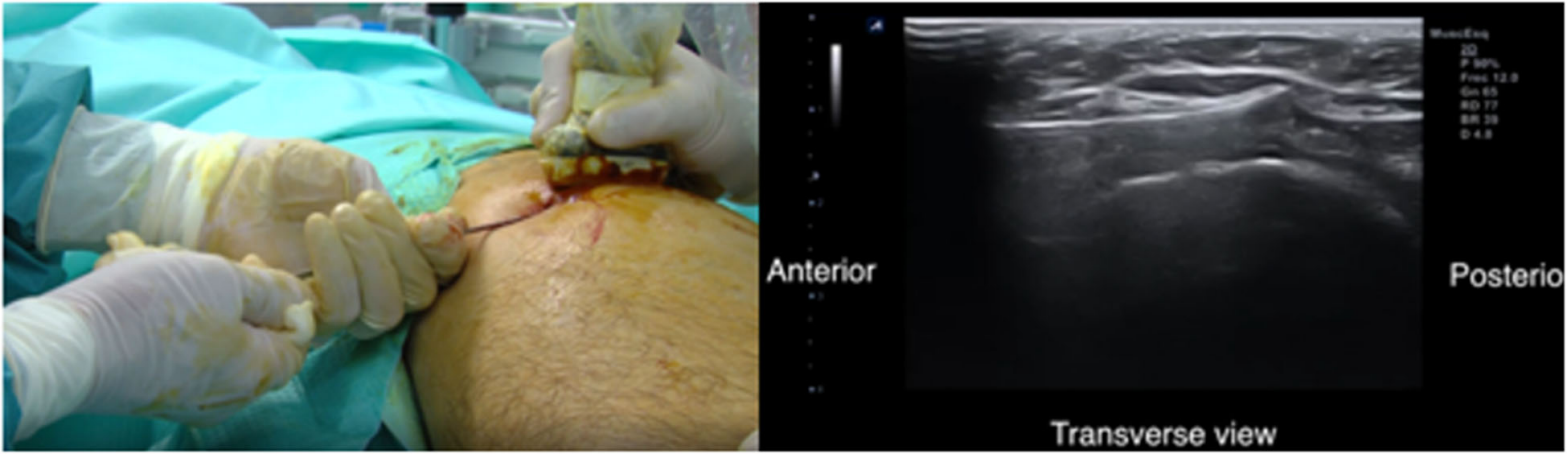
The blunt dissector moves freely from deep to superficial. There is no tension in the ITB

We use the blunt dissector to ensure there is no tension in the tendon and the cut is complete (Fig. 8). Release of the ITB can be assessed directly by measuring the gap between the ends of the tendon.

As the anesthesia is local, we ask the patient to move actively, thus enabling us to check that the snap has disappeared. In cases of doubt, we repeat the surgical gesture. Immediately after surgery, we ensure that there is no tension in the full range of motion, the Ober test is negative, and the snap has disappeared. No stitches are required. The patient can actively move the leg and walk immediately after surgery aided by 1 or 2 crutches.

### Rehabilitation protocol

We encourage active flexion and extension of the hip and knee immediately after surgery, and patients are allowed to walk with 1 or 2 elbow crutches. The crutches are removed as tolerated and are usually no longer necessary after 2–3 days. Patients can also start physiotherapy with passive, active, and stretching exercises for 1 to 2 months. In 1–2 weeks, they start with swimming and isometric exercises, and in 3–4 weeks with cycling and exercises to restore proprioception. Patients can run after 4–6 weeks, although complete recovery may take 8–12 weeks.

## Results

Our preliminary study in 10 cadavers showed the surgical procedure to be safe and effective, with complete release of the proximal ITB from the border of the tensor fasciae latae to the anterior border of the gluteus maximus muscle and no damage to any other structure (Table 1).

**Table 1:** VAS and HHS at 1 and 2 years

Sex/age	Cause	VASpreop	PreopHHS	VAS 3m	HHS 3m	VAS 6m	HSS 6 m	VAS 1 year	HHS 1 year	VAS 2years	HHS 2 years	Satisfaction
Female/29	UK/OU	8	72	0	100	0	100	0	100	0	100	10
Female/39	UK/OU	6	64	0	96	0	100	0	100	0	100	9
Male/44	UK/OU	5	59	0	96	0	100	0	100	0	100	9
Female/44	UK/OU	8	63	0	96	0	96	0	96	0	96	9
Male/42	GF	9	53	4	70	2	76	1	86	1	86	7
Male/45	UK/OU	8	56	1	76	1	96	1	96	1	96	8
Female/34	UK/OU	6	57	0	86	0	100	0	100	0	100	9
Female/38	UK/OU	6	57	0	96	0	100	0	100	0	100	10
Female/30	UK/OU	5	63	0	93	0	100	0	100	0	100	10
Female/41	UK/OU	7	61	0	96	0	100	0	100	0	100	10
Male/50	UK/OU	8	53	1	83	1	100	1	100	1	100	8
Female/62	PO	9	47	4	64	2	70	2	77	2	77	7
Female/33	UK/OU	6	56	0	93	0	100	0	100	0	100	9
Male/45	UK/OU	6	56	1	86	0	96	0	96	0	96	10

*HHS* Harris Hip Score, *VAS* visual analog scale, *UK/OU* unknown/overuse, *GF* gluteal fibrosis, *PO* previous operation

In the clinical series, we operated on 14 patients (5 males and 9 females). Mean age was 43 years (29–62). We performed complete transverse release of the proximal ITB with a single portal in 13 cases. In 2 cases (runners), where the patients also had lateral knee pain, the procedure was combined with ultrasound-guided simultaneous distal release of the ITB.

The snap resolved in all patients, as verified actively and passively during the surgical procedure. Minimum follow-up was 2 (2–4) years; mean follow-up was 3 years. There were no recurrences in the snap at the most recent check-up.

The Harris Hip Score (HHS) score improved from 58 points (47–72) to 88 points (64–100) at 3 months, 95 (77–100) at 6 months, and 96 at 1 year and 2 years. If we exclude the patients with gluteal fibrosis and periprosthetic fracture, the HSS was 91 at 3 months (76–100) and 99 (96–100) at 6 months and thereafter. We found significant differences in the HHS score (*χ*^2^ (4)=52.037, *p*<0.001), with a large and significant effect size (Kendall’s *W*=0.929; 95% CI [0.861, 0.861]) and an increase of 38.1 (35.1, 40.8) points.

The VAS score for sports activity improved from 7 (5–9) before surgery to 1 (0–4) after 3 months and 0 (0–2) after 1.2 years. Excluding the patients with the gluteal fibrosis and periprosthetic fracture of the hip, the VAS was 0 (0–1) for the remaining patients at 3 months and at the last follow-up. We observed significant differences in the VAS score (*χ*^2^ (4)=50.112, *p*<0.0011), with a large and significant effect size (Kendall’s *W*=0.895; 95% CI [0.842, 0.842]) and a decrease of −6.57 (−7.14, −6.14) points.

We confirmed significant differences between the pre-intervention values and those recorded 3–6 months and 1–2 years later in the VAS score (*p*=0.009) and in the HHS score (*p*=0.01). Furthermore, significant differences in the HHS score were found between 3–6 months and 1–2 years (*p*=0.022).

Four portals were necessary in one patient with gluteal fibrosis. After the first single transverse release proved insufficient to resolve the snapping hip, we performed a second release of the ITB parallel to the first one, although the snap persisted. We then completed the resection with 2 additional portals in order to create a z-plasty. The snap only disappeared when we prolonged the posterior vertical arm of the z-plasty by releasing the ITB from the anterior limit of the gluteus maximus. While this was not our original intention, we were forced to choose between completing a closed release or change our procedure from local anesthesia to epidural anesthesia and open surgery. The patient had previously provided informed consent for both procedures. This patient was pain-free after 3 months, and the snap had resolved. A complete and large z-plasty was not sufficient to resolve the snap, and the patient required an additional longitudinal release at the posterior limit between the gluteus maximus and the proximal ITB. He was limited for sports until 1 year, with pain in the buttock when he tried to progress in his training or running activities.

Patients took pain killers for 1–2 days, except for one woman with GTPS after revision surgery for a periprosthetic fracture of a total hip arthroplasty. In this patient, after lateral plating of the femur and subsequent removal of the plate and cables, she complained of invalidating tension, electric shock pain, burning sensation, and limitation on the lateral side of the hip. A snap was identified with a squatting exercise. Although the tension on the lateral side of the thigh and the snap disappeared, she felt pain in the lateral thigh in the area of the scar left by the previous surgery and was treated with amitriptyline 25 mg/day for 3 months and occasional pain killers for 3–4 weeks. We also injected lidocaine with triamcinolone twice in order to improve her hypersensitivity, which gradually resolved between the third and sixth months. She reported an improvement in pain, burning, and tension in the area of the greater trochanter.

Twelve patients were satisfied after 3 months, including the 2 cases with associated distal release of the ITB, and all of them were satisfied at their last check-up.

### Complications

There were no recurrences of the snap at the last check-up, with a minimum follow-up of 2 years. Similarly, there were no complications other than swelling at the surgical site and minor hematomas. Hematomas were larger in the patient with gluteal fibrosis. The late recovery of the patient with gluteus fibrosis may be related to a more exhaustive and deeper release rather to the surgical procedure itself.

## Discussion

GTPS encompasses a range of causes of lateral hip pain including greater trochanteric bursitis, tendinopathy and tears of the gluteus minimus and medius, and LSH. Conservative treatment is successful in more than 90% of cases [3–6, 9]. Lateral hip pain associated with disease of the proximal ITB is often misdiagnosed and attributed to other intra- and extra-articular structures.

LSH syndrome is associated with a tight ITB. Surgical options for LSH range from open surgery—including resection of the ITB, either in combination or not with partial reconstruction of the posterior flap (70–90% success rate) [11–14] and step cut, and z-plasty lengthening (30–100% success rate) [15–21]—to endoscopic surgery. Endoscopic surgery techniques vary from creating a cross-shaped cut plus a diamond defect [22, 23] a transversal release [24] to gluteus maximus tendon release [25].

No surgical techniques have proven superior to transverse release. In fact, open procedures including z-plasty and resection (elliptical, cross-shaped, diamond-shaped) plus partial reconstruction seem to result in slightly poorer outcomes than resections of the fascia or lengthening in terms of recurrence and scar sensitivity [12, 14]. Therefore, effective ultrasound-guided release of the ITB with a 1-mm incision may be a valid and attractive alternative to open or endoscopic surgery, as in other forms of ultrasound-guided surgery.

We postulated that performing complete release of the ITB via a 1- to 2-mm incision was sufficient to interrupt its tension and therefore the snap over the greater trochanter. Our preliminary study in cadavers showed the surgical procedure to be safe and effective, with complete release of the proximal ITB.

During the past 6 years, we have operated on 14 patients following these principles. Our limited experience is promising. Twelve patients were satisfied after 3 months, including the 2 cases with associated distal release of the ITB, and all were satisfied after 1 year.

The patient with severe gluteal fibrosis, who needed several portals and a modification of the technique in order to resolve the snap, experienced pain and was limited for sports for 1 year. We had to perform a large z-plasty and detach part of the union of the gluteus maximus to the ITB using a vertical cut. This may have led to instability, although we may also have damaged part of the gluteal fibers, as the tissue was fibrotic and the snap persisted. Consequently, the patient experienced weakness while running that took 1 year to resolve. However, we do not know whether this is a limitation for ultrasound-guided surgery or simply a special situation that required prolonged training and recovery [37].

The patient with the hip prosthesis reported improvement in pain, burning, hypersensitization, and tension in the greater trochanter area. The snap disappeared. This patient only walks and needs a crutch for long distances.

Of greater interest, despite the delayed functional recovery, is the fact that the snap had not recurred at her last check-up, after a minimum follow-up of 2 years. In addition, as anesthesia is local, the surgeon can ask the patient to actively move the entire hip and leg and check the result immediately. This approach enables us to resolve the snap with a transverse cut, reaching the anterior and posterior border of the proximal ITB. Z-plasty and larger release were necessary in only 1 patient. Given that the technique is easy to perform, it can be modified with partial release or z-plasty.

With the approach described here, complications and surgical aggressiveness are minimal. Furthermore, we can combine other procedures, such as double, proximal, and distal release of the ITB, all with local anesthesia.

Although complete recovery may take 3 months, the rehabilitation protocol is fast and painless in uncomplicated cases (with a VAS of 0 and an HHS of 91 at 3 months). Weight bearing is immediate, and patients usually need crutches for only 2–3 days.

All but 1 patient was satisfied after 3 months, and all were satisfied at their last follow-up. Minor hematomas were the most common complication. No stitches are required, and the incision measures 1–2 mm. Since the risk of bleeding is minimal and immediate weight bearing is allowed, patients did not take low-molecular-weight heparin.

The learning curve is quick, although the surgeon must perfect the technique with cadavers and become competent in the use of ultrasound. This technique is easier than other ultrasound-guided procedures such as tarsal tunnel release, carpal tunnel release, and gastrocnemius lengthening, in which the authors also have experience. However, the technique may be subject to limits and contraindications. First, it may not be possible in cases with gluteal fibrosis, which could make the procedure more complicated and aggressive. Weakness of the abductor muscles with a positive Trendelenburg sign may also be a contraindication [28].

More prospective studies are necessary to compare our approach with other forms of surgery. However, we think ultrasound-guided release of the proximal ITB is an excellent surgical option for LSH, with encouraging results in patients for whom conservative protocols have failed.

## Data Availability

The materials described in the manuscript, including all relevant raw data, are available from the first author upon request by e-mail.

## Abbreviations

LSH: Lateral snapping hip
ITB: Iliotibial band
VAS: Visual analogy scale
HHS: Harris Hip score
GTPS: Greater trochanteric pain syndrome
